# Supplementary material for: CONSORT 2010 Statement: Updated Guidelines for Reporting Parallel Group Randomised Trials
Source: PLoS Med. 2010 Mar 24;7(3):e1000251. doi: 10.1371/journal.pmed.1000251 (PMC2844794; doi:10.1371/journal.pmed.1000251)
Supplement: Supplementary file 2 [file pmed.1000251.s001.doc]

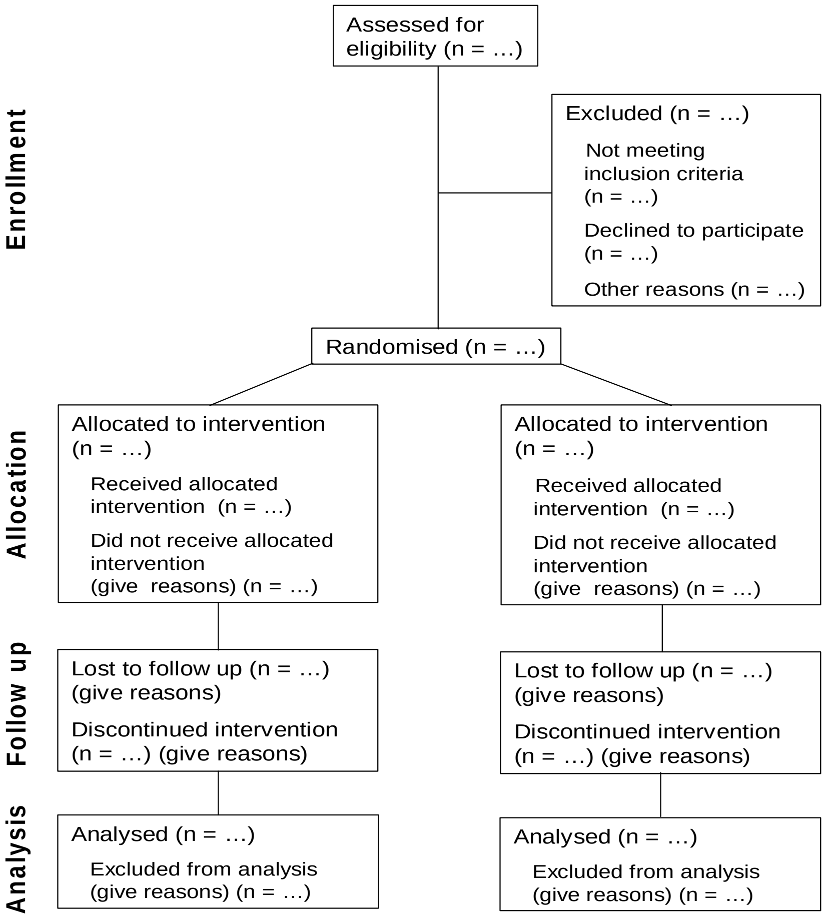
Assessed for eligibility (n = ...)
Enrollment
Excluded (n = ...)
Not meeting inclusion criteria (n = ...)
Declined to participate (n = ...)
Other reasons (n = ...)
Randomised (n = ...)
Allocation
Allocated to intervention (n = ...)
Received allocated intervention (n = ...)
Did not receive allocated intervention (give reasons) (n = ...)
Allocated to intervention (n = ...)
Received allocated intervention (n = ...)
Did not receive allocated intervention (give reasons) (n = ...)
Follow up
Lost to follow up (n = ...) (give reasons)
Discontinued intervention (n = ...) (give reasons)
Lost to follow up (n = ...) (give reasons)
Discontinued intervention (n = ...) (give reasons)
Analysis
Analysed (n = ...)
Excluded from analysis (give reasons) (n = ...)
Analysed (n = ...)
Excluded from analysis (give reasons) (n = ...)
